# Pleckstrin Homology Domain of Akt Kinase: A Proof of Principle for Highly Specific and Effective Non-Enzymatic Anti-Cancer Target

**DOI:** 10.1371/journal.pone.0050424

**Published:** 2012-11-26

**Authors:** Eun-Ha Joh, Joseph A. Hollenbaugh, Baek Kim, Dong-Hyun Kim

**Affiliations:** 1 Department of Pharmacy, Kyung-Hee University, Seoul, South Korea; 2 Department of Microbiology and Immunology, University of Rochester Medical Center, Rochester, New York, United States of America; H. Lee Moffitt Cancer Center & Research Institute, United States of America

## Abstract

While pharmacological inhibition of Akt kinase has been regarded as a promising anti-cancer strategy, most of the Akt inhibitors that have been developed are enzymatic inhibitors that target the kinase active site of Akt. Another key cellular regulatory event for Akt activation is the translocation of Akt kinase to the cell membrane from the cytoplasm, which is accomplished through the pleckstrin homology (PH) domain of Akt. However, compounds specifically interacting with the PH domain of Akt to inhibit Akt activation are currently limited. Here we identified a compound, lancemaside A (LAN-A), which specifically binds to the PH domain of Akt kinase. First, our mass spectra analysis of cellular Akt kinase isolated from cells treated with LAN-A revealed that LAN-A specifically binds to the PH domain of cellular Akt kinase. Second, we observed that LAN-A inhibits the translocation of Akt kinase to the membrane and thus Akt activation, as examined by the phosphorylation of various downstream targets of Akt such as GSK3β, mTOR and BAD. Third, in a co-cultured cell model containing human lung epithelial cancer cells (A549) and normal human primary lung fibroblasts, LAN-A specifically restricts the growth of the A549 cells. LAN-A also displayed anti-proliferative effects on various human cancer cell lines. Finally, in the A549-luciferase mouse transplant model, LAN-A effectively inhibited A549 cell growth with little evident cytotoxicity. Indeed, the therapeutic index of LAN-A in this mouse model was >250, supporting that LAN-A is a potential lead compound for PH domain targeting as a safe anti-cancer Akt inhibitor.

## Introduction

A long-term cell survival phenotype is established by the sensing of various cellular events, and the mechanisms involved in recognition and delivery of stress signals are highly conserved among mammalian cells. The PI3K/Akt pathway is a central regulatory network that governs the cellular events essential for transcription, cell survival [1], growth [2], differentiation [3], migration [4], metabolism [5], and angiogenesis [6]. The dysregulation of the PI3K/Akt pathway is commonly observed in many human cancers, allowing for long-term survival and outgrowth [7], [8], [9]. Thus, pharmacological inhibitors targeting this pro-survival pathway have been extensively investigated as potential anti-cancer agents [10]. Since Akt is a central regulator that controls the activity of numerous downstream targets through its kinase activity, Akt inhibitors have been the focus of several studies [10], [11], [12]. However, most of the Akt inhibitors that have been tested mainly target the kinase active site or ATP binding site of Akt [13], [14], [15], [16] and exhibit potential unwanted off-target effects for numerous other cellular kinases.

Importantly, for Akt kinase to become activated, the protein needs to migrate from the cytoplasm to the cell membrane where the NH_2_-terminal pleckstrin homology (PH) domain of Akt interacts with PI3K. Once at the plasma membrane, constitutively active 3-phosphoinositide-dependent kinase 1 (PDK1), an upstream kinase, activates Akt by phosphorylation at Thr^308^ followed by an additional phosphorylation at Ser^473^, which can occur by mTOR-rictor complex [17], protein kinase Cβ [18], integrin-linked kinase [19] and by autophosphorylation [20]. The PH domain can be found in several intracellular signaling proteins and is need to travel to various cellular membrane compartments [21]. This domain also facilitates dimer formation allowing for the lipid binding feature that recognizes specifically phosphorylated phosphoinositides [22]. During PI3K/Akt activation, PIP2 is phosphorylated to PIP3 by PI3K, and then the elevated PIP3 membrane concentration initiates the activation of PDK1 followed by the membrane translocation of Akt and activation of Akt kinase activity [22].

Various cancer cell models and cells expressing oncogenes, which exhibit a cytoprotective phenotype via activation of the PI3K/Akt pathway, have been used as screening systems for potential Akt inhibitors [23], [24], [25]. We recently established a unique cell-based anti-PI3K/Akt inhibitor screening system [26], which employs the expression of non-oncogenic human immunodeficiency virus (HIV-1) Tat. Unlike other viral oncogenes such as E1A of human papilomavirus [27], Tax of human T cell leukemia virus [28] and NS5A of hepatitis virus C [29], HIV-1 Tat does not directly activate the Akt pathway. Instead, it appears to negatively regulate PTEN, which is a phosphatase that negatively controls PI3K by lowering PIP3 concentration at the cell membrane [30]. Due to PTEN negative regulation activity, Tat expression in a human microglial cell line (CHME5) confers an elevated cell protection phenotype during cytotoxic LPS treatment [31]. This cytoprotective phenotype of the Tat-based CHME5 system was recently used for screening and identified anti-PI3K/Akt compounds that abolished the Tat-induced cytoprotective phenotype [26]. More interestingly, these compounds targeted different steps of the PI3K/Akt pathway, validating the PI3K/Akt pathway inhibitor screening capacity of the system [26].

Here, we characterized lancemaside A (LAN-A), which was one of several compounds previously identified by the Tat expressing CHME5 system from libraries that harbor pre-selected non-toxic compounds [26]. In this study, LAN-A was investigated for its anti-Akt action mechanism, selective anti-cancer effect *in vitro*, and anti-cancer effect in a mouse model. Indeed, LAN-A uniquely binds to the PH domain of cellular Akt kinase, inhibits the translocation of Akt kinase, which is essential for Akt activation, and displays anti-proliferation/anti-cancer effects in a mouse model with a large therapeutic index which results from the non-toxic nature of this compound.

## Materials and Methods

### Cancer cell line culture and measurement of cytotoxicity

The A549 (human lung carcinoma; KCLB No. 10185), KATO III (gastric carcinoma; KCLB No. 30103), MCF-7, (breast carcinoma; KCLB No. 30022) were purchased from the Korea cell line bank (Seoul, Korea) on 2010-11-25. The A549-luc-c8 cell line was purchased from Caliper Lifescience (Hopkinton, MA, USA) on 2011-04-20. All cell lines were grown in RPMI 1640 medium supplemented with 10% heat-inactivated fetal calf serum, 2 mM L-glutamine, and 1 mM sodium pyruvate in a humidified 5% CO_2_ atmosphere at 37°C. Cells were plated in 96-well plates at a density of 1×10^5^ cells per well, 24 h prior to addition of compounds. Compounds were solubilized in 100% ethanol and then added at the indicated concentrations. After 24 h of incubation, dead cells were assessed using FACS cytometry (Accuri C6 flow cytometer, Accuri). All studies were performed in triplicate.

### Co-culturing of primary and tumor cells

To culture different pairs of cell types, the medium of the primary cell type, not the tumor cell line, was used. For co-culturing of human lung fibroblasts and the lung epithelial tumor cell line A549-luc-c8, cells were cultured in minimal essential medium (Invitrogen) containing 10% fetal bovine serum. Images were taken at 0, 6, 12 and 24 h post-treatment using a fluorescence microscope (Zeiss).

### Immunoblot analysis

The cell supernatant extracts prepared from macrophages were separated by 10% SDS-PAGE and transferred to polyvinylidene difluoride membranes (PVDF). The membranes were blocked with 5% non-fat dried-milk proteins in 0.05% PBST, then probed with antibodies. After washing with PBST, proteins were detected with HRP-conjugated secondary antibodies for 50 min. Bands were visualized with enhanced chemiluminescence (ECL) reagent.

### Analysis of Lancemaside A and Akt binding using MS/MS

A549-luc-c8 cells were cultured in 6-well plates in RPMI 1640 medium with or without lancemaside A for 2 h. The cells were lysed with 300 µl of lysis buffer per 100-mm culture dish. Lysates (3 ml) were supplemented with 9 ml of NET buffer [50 mM Tris-HCl (pH 7.4), 150 mM NaCl, 5 mM EDTA, and 0.05% NP-40] and incubated overnight at 4°C with 10 µl of Akt or β-actin antibody (200 µg/ml). To precipitate immune complexes, lysates were incubated with 50 µl of protein A/G PLUS-Agarose (Santa Cruz, L.A., U.S.A.) for 1 h at 4°C. Bead-bound complexes were washed three times with cold NET buffer and denatured for 5 min at 100°C, centrifuged and detected using MS/MS system as previously described [32]. Electrospray ionization mass spectrometry (ESI-MS) and tandem MS/MS analyses were performed on a LCQ DECA XP MS (Thermo Finnigan, CA, USA) equipped with an electrospray ion source. All ion trap analyzer parameters were optimized according to the manufacturer's instructions. In mass experiments, the spray voltage was 4.5 kV in positive mode and 4 kV in negative mode under N_2_ sheath gas flow at 50 arbitrary units. The capillary temperature was maintained at 275°C. Two microliters of samples were injected into the column. Total ion chromatograms from *m/z* 150 to 2000 in ESI negative mode were obtained. For tandem mass spectrometry, the maximum ion injection time, activation time, and isolated ion width were set to 500 ms, 30 ms and 2.0 Th, respectively. The collision energy with helium was set to 30% of the radio frequency (5 V) applied to the ion trap analyzer.

### Anti-tumor activity in the nude mouse tumor xenograft model

A549-luc-c8 cells were harvested, resuspended in phosphate-buffered saline, and injected subcutaneously into the left leg (10^6^ cells/leg) of 8-week old female nude mice as reported previously [33]. When tumors reached about 50 mm^3^, animals were randomized and orally dosed with 5, 10 and 20 mg/kg/day lancemaside A or vehicle (0.2 ml of 10% TWEEN 80) 6 days per week. Each group consisted of 9 mice. Lancemaside A was suspended in 10% TWEEN 80. The tumor sizes were measured every 3–4 days after reaching 50 mm^3^. Finally, we intraperitoneally injected luciferin reagent (150 mg/kg, Caliper Lifescience) after the final administration of lancemaside A and measured tumor sizes by *in vivo* luminescence imaging. *In vivo* luminescence imaging was performed using Maestro In Vivo Multispectral Imaging System (Woburn, MA, USA). Data were quantified with Maestro software (version 2.10.0, CRi Inc.) by using absolute photon counts.

### Human A549 cells transfected with PH-GFP or GFP vectors

The A549-luc-c8 cells were transfected with PH-GFP or GFP vector and were cultured in 6-well plates in RPMI 1640 medium with or without lancemaside A (1 µM) for 100 min. The cells were lysed with 300 µl of lysis buffer per well. Lysates were supplemented with 5 ml of NET buffer [50 mM Tris-HCl (pH 7.4), 150 mM NaCl, 5 mM EDTA, and 0.05% NP-40] and incubated overnight at 4°C with 10 µl of GFP antibody (200 µg/ml). To precipitate immune complexes, lysates were incubated with 50 µl of protein A/G PLUS-Agarose (Santa Cruz, L.A., U.S.A.) for 1 h at 4°C. Bead-bound complexes were washed three times with cold NET buffer and denatured for 10 min at 100°C before being centrifuged to remove debris. Supernatants were evaporated to dryness and resuspended in 50 µl of MeOH.

## Results

**Figure 1:**
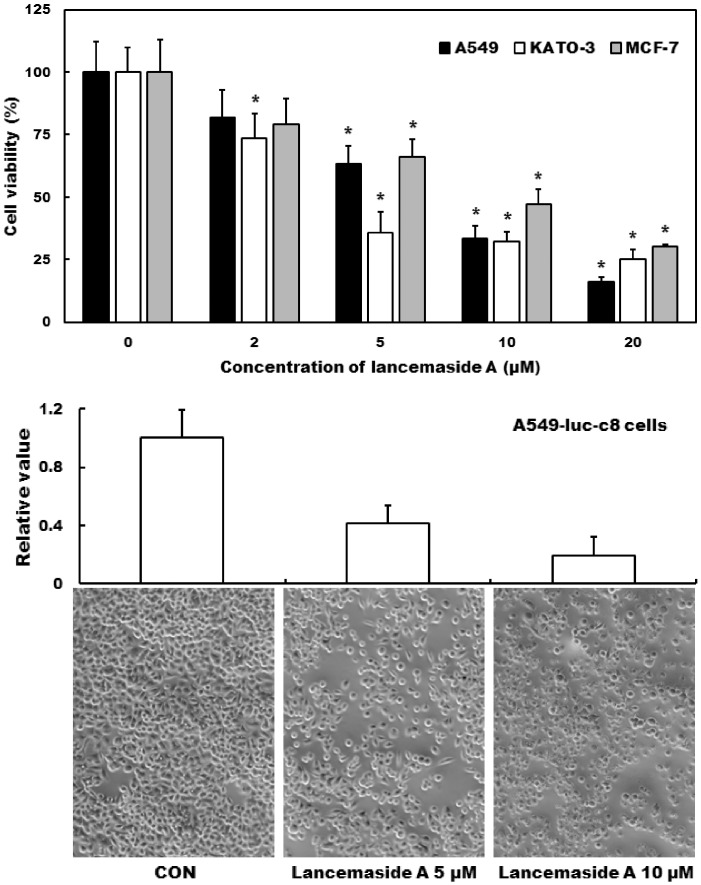
Cytotoxicity of Lancemaside A in different cell lines. (**A**) A549, KATO III and MCF-7 cells were treated for 24 h in the absence or presence of different concentrations of LAN-A. After treatment, cells were collected and live cells were determined by FACS analysis. (**B**) A549-luc-c8 cells were treated with 5 and 10 µM LAN-A for 24 h. Images show less confluent monolayer of cells with LAN-A treatment. Cell viability was determined and relative values plotted as compared to that of the control cells (100%). ANOVA analysis was performed on data sets, and statistically significant differences from the control group are indicated with asterisks (* = *p*<0.05).

We previously had identified LAN-A as an anti-PI3K/Akt pathway inhibitor using a HIV Tat expressing CHME5 cell line [26]. We, first, examined the ability of LAN-A to inhibit this pathway in several different tumor cells. As shown in **Figure 1A**, increasing concentrations of LAN-A reduced cell survival in A549 (adenocarcinoma epithelial cell), KATO III (gastric carcinoma cell) and MCF-7 (breast carcinoma cell) cells in a dose dependent manner, leading to significant differences as compared to control (untreated) cells with as little as 2 µM of drug. At 20 µM LAN-A, the highest concentration used, greater than 60% reduction in viability was detected for all three cell lines. Furthermore, western blot analysis confirmed the reduction in pAKT levels for the three cell lines (**Figure S1**). The A549-luc-c8 cells were also used for this study and we confirmed that LAN-A treatment also caused a comparable reduction in cell viability (**Figure 1B**). A549 cells have a round morphology as shown in **Figure 1B** bottom panels, which is an important consideration when examining the co-culture experiments (below).

**Figure 2:**
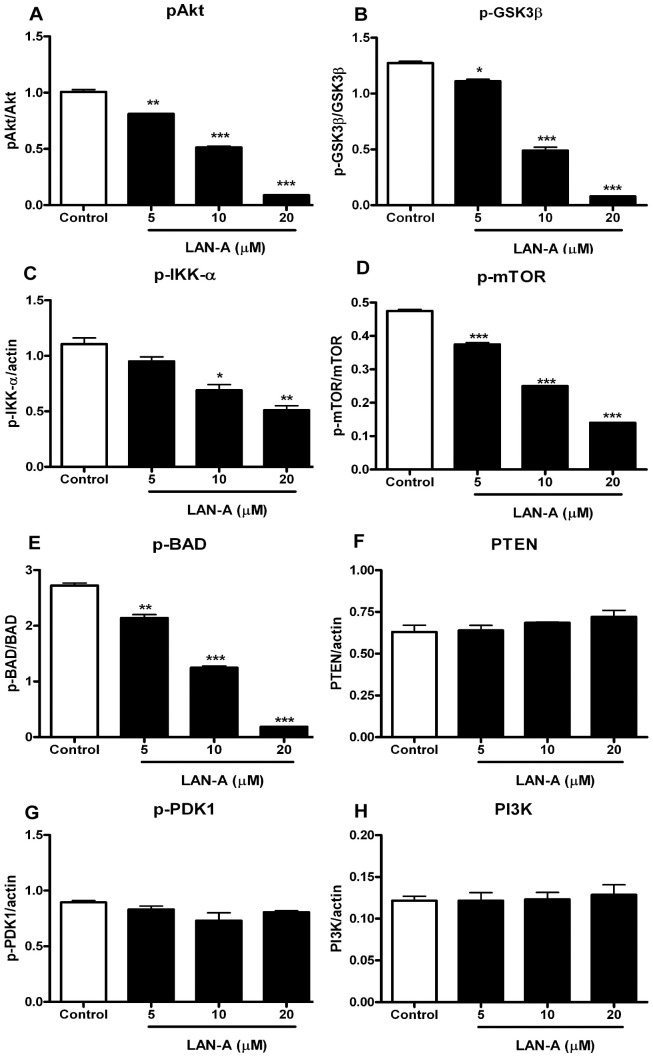
Western blot analysis of LAN-A treated A549 cell lysates. (**A**) A549 cells were left untreated or treated with 5, 10 or 20 µM LAN-A for 24 h. Western blot analysis was conducted on cell lysates (**Supplementary** **Figure 2**) and indicated a reduction in phosphorylated Akt (Ser^473^). (**B–E**) Analysis of Akt downstream targets is shown. The phosphorylation levels for GSK3β, IKK-α, mTOR and BAD were examined and are reduced in LAN-A treated groups. (**F**) Cellular PTEN remained constant with LAN-A treatment. (**G**) Phospho-PDK1 level remained unchanged with LAN-A treatment. (**H**) PI3K level remained unchanged with LAN-A treatment. Data sets were done in duplicate. ANOVA analysis was performed on the data sets with * = *p*<0.05, ** = *p*<0.01 and *** = *p*<0.001.

To further validate LAN-A as a PI3K/Akt pathway inhibitor in A549 cells, western blot analysis was conducted for the phosphorylation status of various downstream targets: PDK1, Akt, PTEN, GSK3β, IKK-α, mTOR and BAD proteins. As shown in **Figure 2A–E** (and **Figure S2**), phosphorylation of Akt and downstream targets of Akt kinase: p-GSK3β, p-mTOR, p-BAD and p-IKK-α were significantly reduced in a dose dependent manner with LAN-A treatment. However, LAN-A treatment did not lead to reduction in cellular levels of PTEN (**Figure 2F**), p-PDK1 (**Figure 2G**) or PI3K (**Figure 2H**). Moreover, LAN-A reduced PI3K activity in A549 cells (**Figure S3**) and inhibited Akt trafficking to the plasma membrane (**Figure S4**). These results are consistent with our published findings [26] that LAN-A is a PI3K/Akt pathway inhibitor.

**Figure 3:**
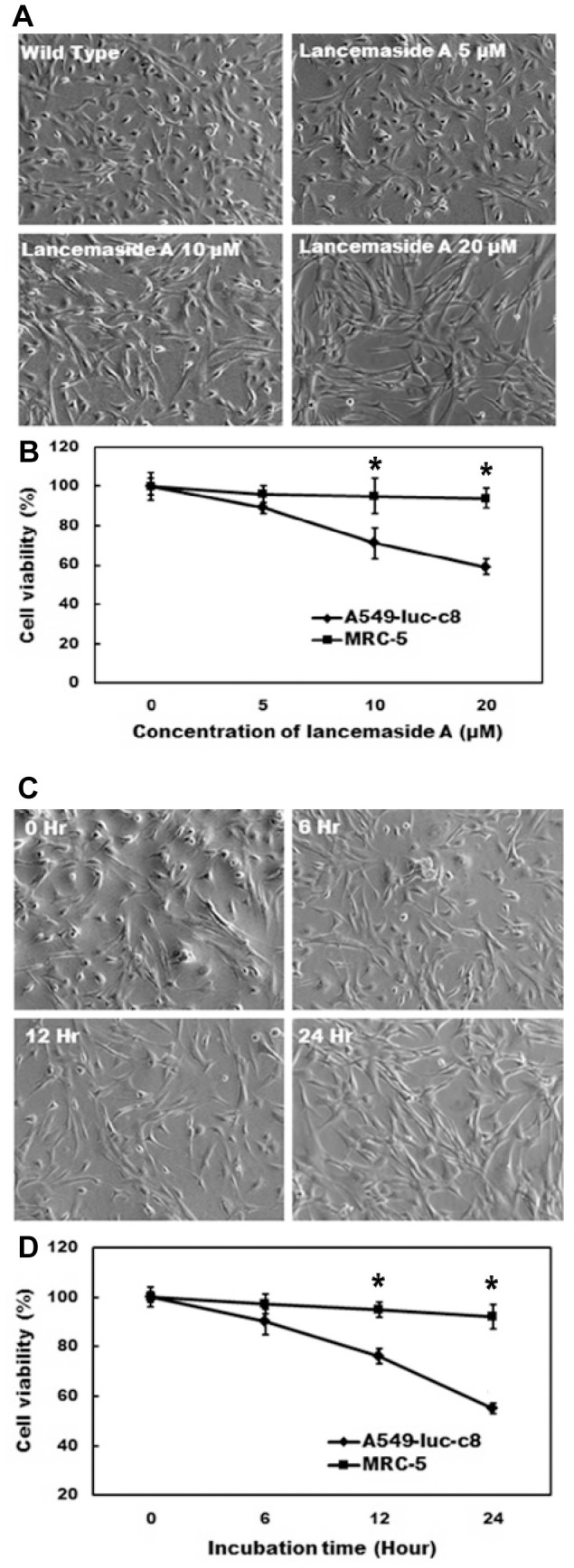
The cytotoxicity of LAN-A against co-cultured MRC-5 and A549-luc-c8 cell lines. (**A**) Representative images for the different treatment groups were captured 12 h post treatment. (**B**) Images from three independent experiments were manually counted for the live/dead cells for the different treatment groups. Data were plotted and shows the percentage of live cells. Student's T test was performed on the data sets to determine significant differences (* = *p*<0.05). (**C–D**) Co-cultured cells were exposed to 10 µM LAN-A and then monitored for cell death at different times post treatment. Cell death was determined as described above. Data are from three independent experiments.

Next, we tested whether the LAN-A cytotoxic effect was specific for tumor cells. For this test, we co-cultured 1) MRC-5, a fetal primary human lung fibroblast [34], and 2) A549-luc-c8, adenocarcinoma human alveolar basal epithelial cells transduced to express luciferase [35], under different concentrations of LAN-A for 12 h (**Figure 3A** **, top**). MRC-5 cells remained viable over the dose range of LAN-A treatment (**Figure 3A** **; bottom graph**), whereas the A549-luc-c8 cells shown significant death at 10 and 20 µM. A kinetic analysis using 10 µM LAN-A treatment at 0, 6, 12 and 24 h was also conducted (**Figure 3B** **, top**). Again, A549-luc-c8 cells were significantly more susceptible to cell death as compared to MRC-5 cells (**Figure 3B** **, bottom graph**). Even after 24 h of LAN-A treatment, MRC-5 cells remained viable whereas A549-luc-c8 cell viability continued to decline. Collectively, these data suggest that LAN-A displays a specific anti-cancer effect in the human lung normal and cancer cell co-culture model.

**Figure 4:**
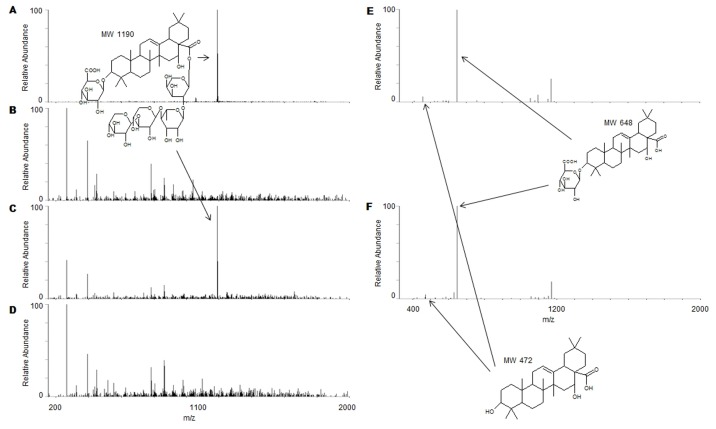
Mass spectrometry analysis of LAN-A interaction with Akt. (**A**) Electrospray ionization mass spectrometry (ESI-MS) of LAN-A standard shown. LAN-A molecular is shown to reside at MW 1190. ESI-MS analysis of IP Akt samples from untreated (**B**) LAN-A treated (**C**) A549 cells are shown. (**D**) ESI-MS analysis of IP β-actin from LAN-A treated cells is shown. (**E**) Tandem MS/MS analysis of LAN-A shows the two previously identified fragments Lancemaside X (MW 648) and echinocystic acid (MW 472) for LAN-A [32]. (**F**) Tandem MS/MS analysis for IP Akt sample from LAN-A treated cells.

Next, we investigated the action mechanism of LAN-A employing a series of mass spectra technologies. For this, A549 cells were pre-incubated with LAN-A before lysing and immunoprecipitation (IP) of Akt. Pull-downs were analyzed using mass spectral analysis. **Figure 4A** shows the ESI-MS analysis of LAN-A (standard). The compound structure is depicted and a trace profile with LAN-A at MW 1190 is provided. An IP Akt sample for LAN-A treated A549 cells was analyzed (**Figure 4C**), and showed a peak for LAN-A. Importantly, the control samples, no LAN-A treatment of A549 cells (**Figure 4B**) and β-actin IP with LAN-A treatment (**Figure 4D**), did not show peaks for LAN-A interaction. Next, to further validate that LAN-A interacted with Akt, tandem MS/MS analysis was done. We previously developed a sensitive HPLC-MS/MS analysis for LAN-A (MW 1190) and its metabolic derivatives, lancemaside X (MW 648) and echinocystic acid (MW 472) [32]. The standard trace profile of LAN-A only is shown in **Figure 4E**, while IP Akt sample clearly shown the two fragments – MW 648 and MW 472 (**Figure 4F**). These data clearly show that LAN-A physically interacts with Akt within the cell.

**Figure 5:**
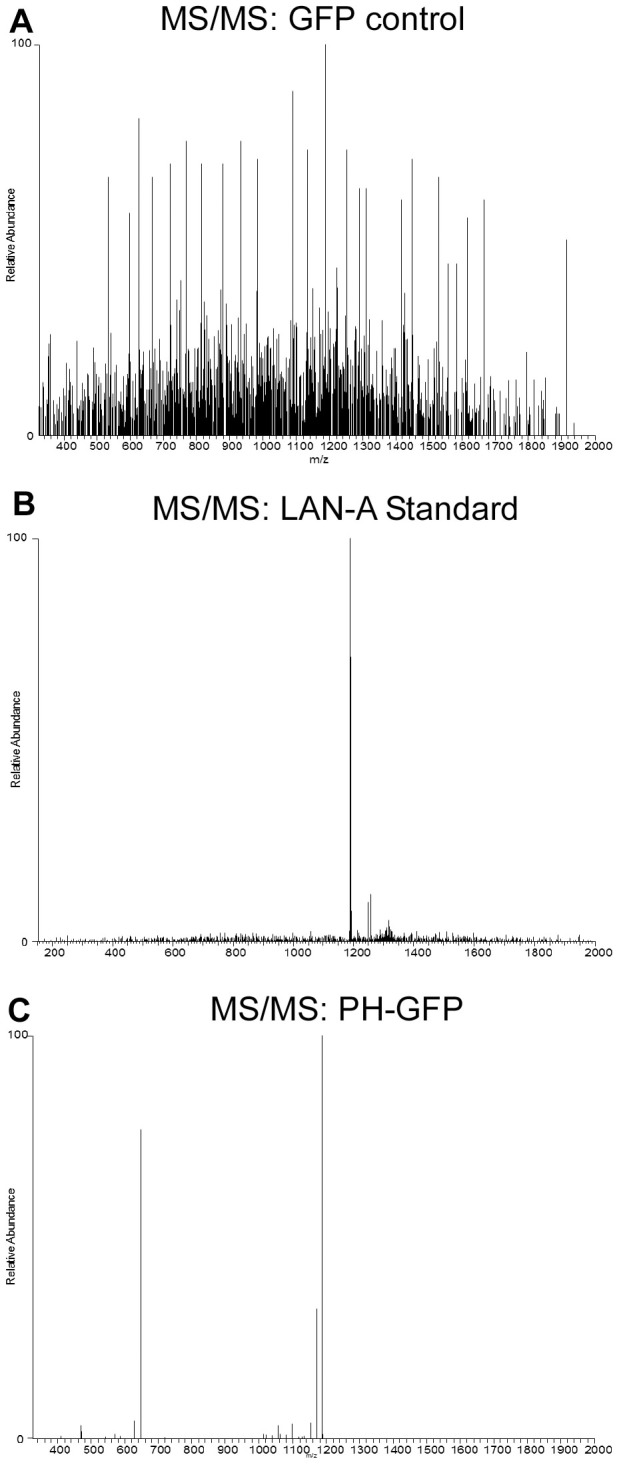
LAN-A interacts with the PH domain of Akt. GFP and PH domain-GFP vectors were transfected into A549-luc-c8 cells and cells were treated with 100 µM LAN-A for 1 h. IP against GFP was done and samples were analyzed by ESI-MS. (**A**) IP GFP sample showed no prominent peak for LAN-A, (**B**) whereas IP PH domain-GFP sample had the specific peak for LAN-A. (**C**) To further confirm this was LAN-A, tandem MS/MS analysis of the IP PH domain-GFP sample showed two fragments for LAN-A (also see **Figure 4E** and **F**).

We have previously described that LAN-A inhibits translocation of Akt from the cytosol to the plasma membrane ([26]; **Figure S4**). Therefore, we postulated that LAN-A directly interacts with the PH domain of Akt. To test this, PH domain-GFP fusion and GFP vectors were transfected into A549 cells, followed by IP of GFP. Samples were then processed for and analyzed using HPLC-MS/MS [32]. The IP GFP sample (**Figure 5A**) produced many peaks, but none were specific for LAN-A (**Figure 5B**; standard only). However, MS/MS analysis of IP PH-GFP sample produced the correct metabolite derivatives peaks (MW 648 and MW 472) for LAN-A (also see **Figure 4E and F**). Therefore, these mass spectra data support a model in which LAN-A directly interacts with PH domain of Akt, which restricts Akt migration and subsequently inhibits Akt activation.

**Figure 6:**
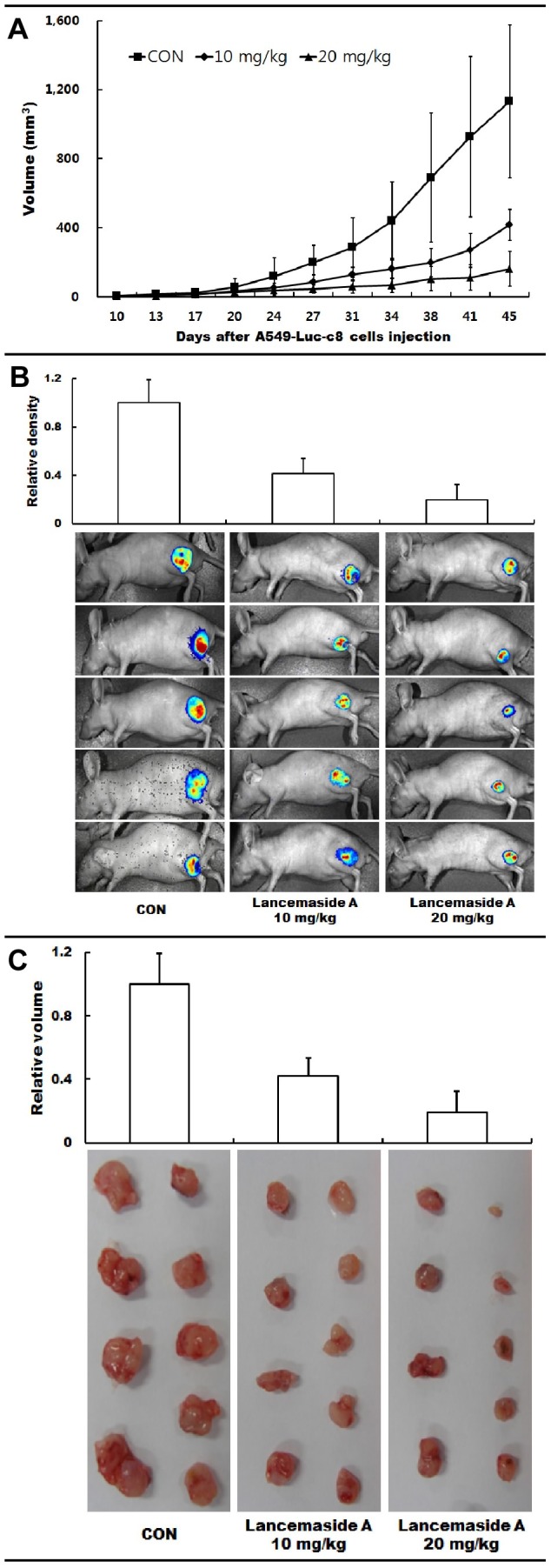
Effects of LAN-A on the growth of A549-luc-c8 cells *in vivo*. (**A**) Tumor volumes were measured from days 10–45 in nude mice treated with control (con) and 10 or 20 mg/kg/day LAN-A (9 mice/group). (**B**) At day 45, mice were injected with luciferin reagent for luminescence live imaging. Relative intensities for 5 mice/group are shown, and the relative intensity was indicated compared to that of the control (control = 1). (**C**) Mice were sacrificed and tumors removed. Tumors were measured by Maestro *In Vivo* Multispectral Imaging System and relative volume plotted. ANOVA analysis was performed on data sets, and statistically significant differences from the control group are indicated with asterisks (* = *p*<0.05).

Finally, we tested the effect of LAN-A on A549-luc-c8 growth in a nude mouse model. For this test, A549-luc-c8 cells were injected into the left leg of nude mice to establish tumors. Once the tumor reached 50 mm^3^, mice were randomized and treated orally with 10 and 20 mg/kg/day lancemaside A or vehicle (10% TWEEN 80) 6 days per week. LAN-A has an ED_50_ = 4.09 mg/kg and LD_50_ = >1 g/kg. Therefore, the therapeutic index is >250 (data not shown). Tumors were measured from days 10 to 45. LAN-A treatment tumor reduced growth rate as depicted by reduced tumor volumes as compared to the control group (**Figure 6A**). At day 45, mice were IP injected with luciferin reagent for *in vivo* imaging (**Figure 6B**). *In vivo* luminescence is greater for the control group as compared to either the 10 or 20 mg/kg LAN-A groups. The relative intensities of luminescence are shown (*n* = 5). The mice were sacrificed and tumors removed. Relative volumes were determined and were plotted (*n* = 9) for tumor mass (**Figure 6C**). Tumors were then stained with anti-pAkt and DAPI (**Figure S5**) and displayed a LAN-A concentration dependent reduction of pAkt within the tumor tissue. Overall, we conclude that LAN-A has an anti-tumor effect *in vivo*.

## Discussion

Previously, we screened potential anti-cancer agents, which exhibited minimal cytotoxicity and high natural abundances, by employing the strong cytoprotective phenotype induced by the expression of HIV-1 Tat protein [26], which was observed in both primary human macrophages and a human microglia cell line [30], [31]. Among the screened compounds, treatment with LAN-A abolished the Tat-induced cytoprotective phenotype of CHME5 cells after LPS/CHX treatment, while LAN-A alone did not induce any cell death in the CHME5 control cells not expressing Tat [26]. LAN-A belongs to the chemical family called saponins, which are known to exhibit anti-inflammatory and anti-tumor properties [33], [36], [37], [38]. LAN-A has been shown to potently inhibit colitis via TLR-linked NF-κB activation in mice with no detectable cytotoxicity [39], [40].

In this study we further characterize the anti-cancer effect of LAN-A. Co-culture experiments using A549 and MRC-5 cells show that LAN-A specifically inhibits the tumor cell line and not primary cells. This was a very exciting result and suggested that LAN-A had a cancer cell specific effect. We show that treatment of cells with LAN-A leads to a reduction in phosphorylation of Akt, yet p-PDK1, which is required for Thr^308^ phosphorylation, and total PDK1 levels were not influenced by LAN-A treatment. Moreover, downstream targets including p-GSK3β, p-mTOR, p-BAD and p-IKK-α were reduced, while PTEN and PI3K levels remained unchanged (**Figure 2**). Collectively these data support our previous findings that LAN-A inhibits Akt activation [26].

Several different mechanisms for Akt inhibition have been reported. First, drugs like wortmannin, LY294002, PWT-458 [41] and PX-866 [42], [43], SF-1126 [44], IC87114 and TGX-115 [45] target the upstream PI3K effector, inhibiting the generation of phosphatidylinositol 3,4,5-trisphosphate, which activates PDK1 leading to Akt phosphorylation and activation. Other drugs have dual targeting for PI3K and mTORC2, including NVP-BEZ235 [46]. Several drugs have been developed that target PDK1 activity. Unlike PI3K, which has several isoforms, PDK1 has only a single isoform in humans, making it an attractive target for inhibition. UCN-01 is one such drug in development [47]. The second Akt phosphorylation at Ser^473^ is catalyzed by mTORC2 [48] and can be inhibited by rapamycin, temsirolimus, everolimus and deforolimus [49].

Akt has three different isoforms and several different groups of Akt inhibitors have been developed. One group of inhibitors: GDC-0068 (Genetech; [50]) and Akt Inhibitor IV (Millipore) targets the ATP active site of Akt [51]. A second group of inhibitors target the PH domain, leading to disruption of membrane targeting [52], [53]. Akt-I-1/2 are synthetic reversible allosteric inhibitor, which target the PH domain retaining it in an inactive state [54]. Perifosine is another PH domain targeting drug and inhibits Akt, mTORC1 and mTORC2 [55].

To determine the specificity of LAN-A, we previously developed a specific HPLC-MS/MS analysis for LAN-A [32]. Using this technology, we determined that LAN-A directly interacts with Akt through the PH domain (**Figure 5**). We previously have examined oral dosing of mice with LAN-A and found that the intestinal flora converts LAN-A into lancemaside X and then into echinocystic acid, which can be absorbed into the blood [32]. We can postulate from our data that echinocystic acid may be the minimum structure metabolite from LAN-A required for PH domain binding and Akt kinase inhibition. Both lancemaside X and echinocystic acid will be examined for their therapeutic efficacy in the future. Furthermore, our *in vivo* mouse data is consistent with the results of Jo *et al.* [53], showing that targeting the PH domain of Akt kinase is very effective at slowing tumor growth.

There has been extensive investigation to discover inhibitors of the PI3k/Akt/mTORC2 pathway [56], [57]. Many new drugs have to deal with toxicity while providing therapeutic efficacy. LAN-A provides low toxicity and inhibition of tumor growth in vivo. Collectively, our study validates by mass spectral analysis that LAN-A targets the PH domain of Akt and provides a valid approach for continued research on this anti-cancer drug.

## Supporting Information

Figure S1 **Western blot analysis of A549, KATO III and MCF-7 cells.** (**A**) Shown are representative western blots probing for pAkt and total Akt with and without 10 µM LAN-A. (**B**) Data from two independent experiments was graphed. ANOVA analysis was used to determine significant differences and are indicated with asterisks (*** = *p*<0.001).
(TIF)

Figure S2 **Western blot analysis.** This is a composite for one or two western blots analysis used to generate the data for **Figure 2**.
(TIF)

Figure S3 **PIP3 assay.** A549 cells were pre-treated with the lancemaside A (Lan, 20 µM) or wortmannin (W, 0.1 µM) for 20 min. Then, cells were treated with LPS for an additionally 60 min. Lysates were prepared using a compartmental protein extraction kit (Millipore, Bedford, MA, USA) from an equal numbers of the treated cells. The membrane extract lysates were analyzed by western dot blot analysis (Echelon, San Jose, CA, USA) and visualized with anti-PIP3 antibody.
(TIF)

Figure S4 **Assay of Akt membrane translocation.** We expressed a GFP protein fused to the PH-domain of Akt, which is known for the membrane migration, in A549-luc-c8 cells. When cells were transfected with a plasmid expressing the PH-Akt-GFP protein, this fusion protein was observed throughout the cells, but mainly localized at the plasma membrane. LAN-A treatment shows the GFP signal has moved away from the plasma membrane. Bright field (BF) images are on the left column. Akt plasma membrane localization was visualized by the movement of Akt-PH-eGFP using a fluorescence microscope (Zeiss).
(TIF)

Figure S5 **Immunohistochemistry of pAKT within the tumor.** Immunolocalization of pAkt was analyzed using a two-step staining procedure consisting of sequential incubation with primary and secondary antibodies and with DAPI. Images were taken using a fluorescence microscope (Zeiss).
(TIF)
