# Supplementary figures and images for: Pleckstrin Homology Domain of Akt Kinase: A Proof of Principle for Highly Specific and Effective Non-Enzymatic Anti-Cancer Target
Source: PLoS One. 2012 Nov 26;7(11):e50424. doi: 10.1371/journal.pone.0050424 (PMC3506615; doi:10.1371/journal.pone.0050424)

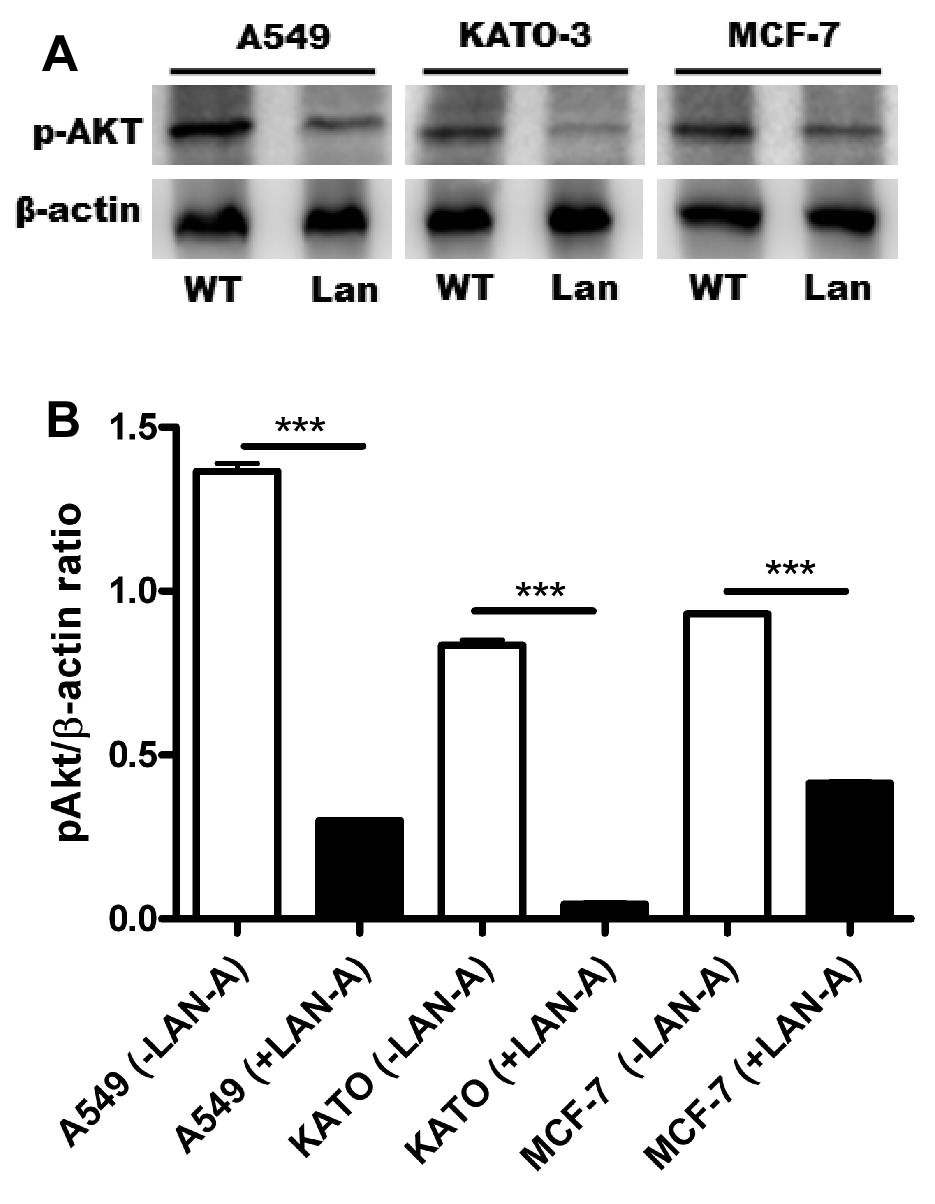

Supplement: Figure S1 — Western blot analysis of A549, KATO III and MCF-7 cells. (A) Shown are representative western blots probing for pAkt and total Akt with and without 10 µM LAN-A. (B) Data from two independent experiments was graphed. ANOVA analysis was used to determine significant differences and are indicated with asterisks (*** = p<0.001). (TIF) [file pone.0050424.s001.tif]

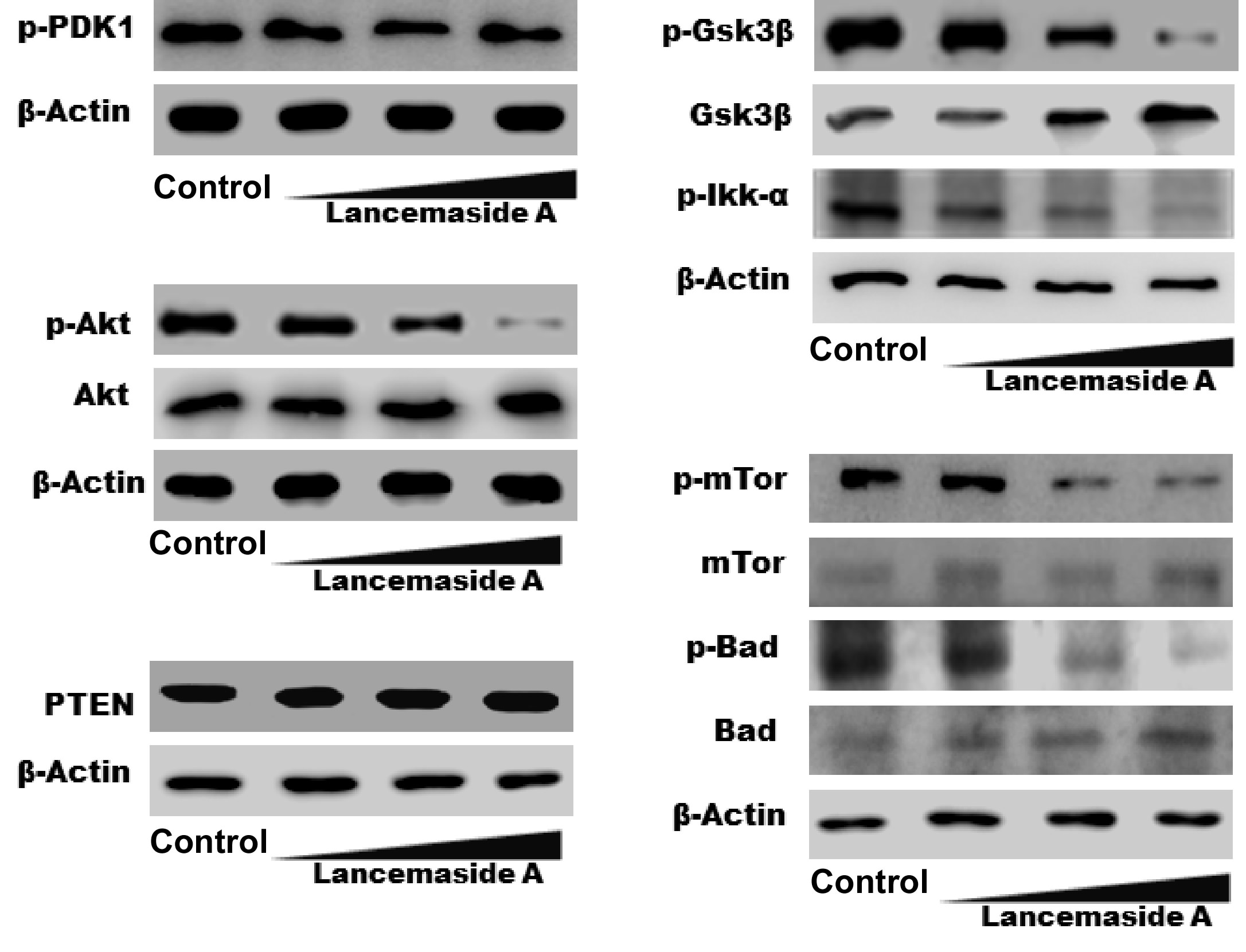

Supplement: Figure S2 — Western blot analysis. This is a composite for one or two western blots analysis used to generate the data for Figure 2 . (TIF) [file pone.0050424.s002.tif]

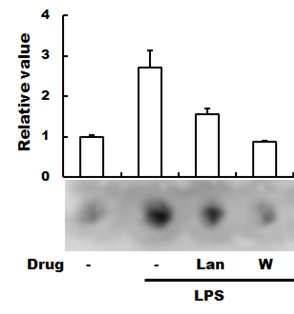

Supplement: Figure S3 — PIP3 assay. A549 cells were pre-treated with the lancemaside A (Lan, 20 µM) or wortmannin (W, 0.1 µM) for 20 min. Then, cells were treated with LPS for an additionally 60 min. Lysates were prepared using a compartmental protein extraction kit (Millipore, Bedford, MA, USA) from an equal numbers of the treated cells. The membrane extract lysates were analyzed by western dot blot analysis (Echelon, San Jose, CA, USA) and visualized with anti-PIP3 antibody. (TIF) [file pone.0050424.s003.tif]

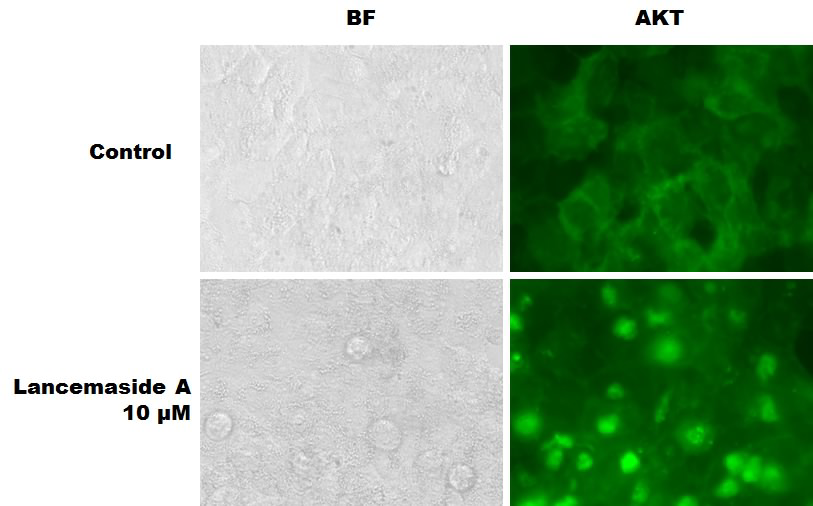

Supplement: Figure S4 — Assay of Akt membrane translocation. We expressed a GFP protein fused to the PH-domain of Akt, which is known for the membrane migration, in A549-luc-c8 cells. When cells were transfected with a plasmid expressing the PH-Akt-GFP protein, this fusion protein was observed throughout the cells, but mainly localized at the plasma membrane. LAN-A treatment shows the GFP signal has moved away from the plasma membrane. Bright field (BF) images are on the left column. Akt plasma membrane localization was visualized by the movement of Akt-PH-eGFP using a fluorescence microscope (Zeiss). (TIF) [file pone.0050424.s004.tif]

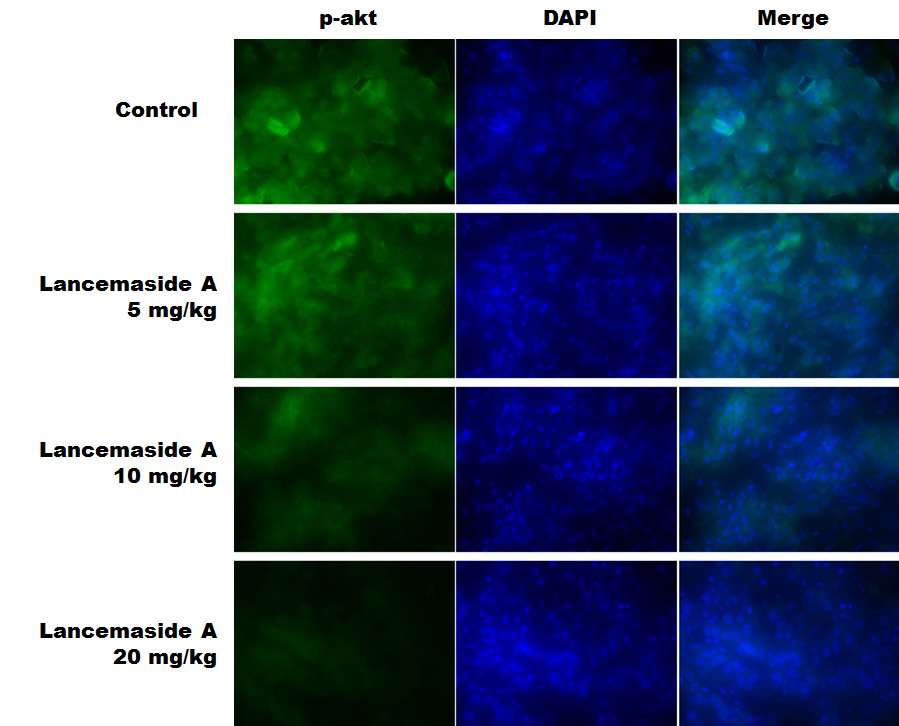

Supplement: Figure S5 — Immunohistochemistry of pAKT within the tumor. Immunolocalization of pAkt was analyzed using a two-step staining procedure consisting of sequential incubation with primary and secondary antibodies and with DAPI. Images were taken using a fluorescence microscope (Zeiss). (TIF) [file pone.0050424.s005.tif]
